# Comparative Age Estimation Using 2D and 3D Imaging of Maxillary and Mandibular Molars: A Prospective Study

**DOI:** 10.7759/cureus.80141

**Published:** 2025-03-06

**Authors:** Ayushi P Mangulkar, Prajakta C Khelkar, Bharathi S, Freny R Karjodkar, Sunali Khanna, Nilam Sonawane, Resham V Pakhmode, Aswathi Unnikrishnan, Shivani Singh, Tanushree Jadhav

**Affiliations:** 1 Oral Medicine and Radiology, Nair Hospital Dental College, Mumbai, IND; 2 Department of Dentistry, Gokuldas Tejpal College and Hospital, Mumbai, IND

**Keywords:** 2d and 3d imaging, 3d slicer software, age estimation, cbct, tooth coronal index

## Abstract

Background

In forensic odontology, age estimation is vital for identification, legal, and investigative purposes. Teeth, resistant to external factors, serve as reliable indicators of biological maturity. The present study aims to assess the accuracy and reliability of age estimation by comparing pulp-to-enamel volume ratios from 3D imaging with the Tooth Coronal Index (TCI) method using panoramic radiographs.

Methodology

This prospective study analyzed 620 radiographs from participants aged 16-65 years, categorized into five age groups. Using random sampling, 320 images each were evaluated using the following two methods: TCI measured from panoramic radiographs and pulp-to-enamel volume ratio using cone-beam computed tomography (CBCT) and 3D Slicer software. TCI was calculated using crown and pulp cavity heights from maxillary first (MX1) and second molars (MX2) and mandibular first (MND1) and second (MND2) molars. CBCT scans were processed in DICOM format for 3D segmentation and analysis of pulp volume (PV) and enamel volume (EV). Statistical analyses were performed using SPSS version 26.0 (IBM Corp., Armonk, NY, USA), employing t-tests, analysis of variance, and regression analysis. A p-value <0.05 was considered statistically significant.

Results

Among 320 teeth analyzed, TCI showed a statistically significant correlation with age only for MX2 (p < 0.05). The standard error of estimate for TCI-based age prediction was 14.57 years, with poor regression equations (R² ≈ 0). PV/EV analysis demonstrated a strong correlation with age (R² > 0.8), with a standard error of 4.34 years. Linear regression equations derived for PV/EV provided reliable age predictions. MX1, MX2, and MND2 PV/EV showed a significant positive correlation with age (p < 0.01), with R² nearing 1 for individual predictors. The youngest subject was 16 years old, and the eldest was 64 years old.

Conclusions

Considering certain limitations, this study concluded that 3D imaging, particularly utilizing maxillary and mandibular molars, demonstrates greater accuracy in age estimation compared to 2D imaging and shows significant potential for forensic applications.

## Introduction

Age is a crucial factor in forensic medicine and dentistry for identification, especially when external appearance is misleading [1]. It aids in crime investigations, legal cases, immigration cases, and assessment of the medicolegal importance of age in both living and deceased individuals [2,3]. Growth involves changes in physiological systems, including the skeleton and dentition, leading to maturity reflected in teeth [4]. Human dentition, unique like fingerprints, is a reliable indicator of biological maturity [5]. Forensic odontologists often estimate age using teeth, as they are less influenced by nutrition or hormones compared to other systems [6]. In early 19th-century England, Edwin Saunders first introduced the concept of using teeth for age estimation in his pamphlet “The Teeth a Test of Age” (1837), presented to the English parliament [7]. The literature outlines various techniques for adult age estimation such as morphological methods, which assess ex-vivo teeth, including notable contributions by Gustafson, Dalitz, Bang, and Ramm, among others [8].

Biochemical methods focus on the racemization of amino acids, a reversible first-order reaction prominent in slow-metabolizing tissues, with methods developed by Helfman and Bada (1975, 1976) and Ritz et al. (1995) [9]. Radiological methods, introduced by Schuller in 1921, are indispensable in forensic science, being non-invasive and applicable to both the living and deceased [1,8]. The determination of age through dental radiographic methods has been a focus of forensic science, with various approaches and technological advancements improving accuracy. Cameriere et al. (2004) demonstrated a strong correlation between pulp/root ratios in maxillary canines and chronological age, explaining 84.9% of variations, establishing it as a reliable age estimation tool [9]. Bosmans et al. (2005) adapted Kvaal’s method for panoramic radiographs [10], while Shi et al. (2008) and Star et al. (2010) highlighted the role of secondary dentin in age estimation using cone-beam computed tomography (CBCT) [11,12]. Maret et al. (2011) proposed CBCT integration for improved accuracy, supported by Sakuma et al. (2013) and Pinchi et al. (2015) for post-mortem analyses [13-15]. Ge et al. (2015) and Sue et al. (2017) emphasized the utility of CBCT but noted limitations compared to micro-CT technology [16,17].

The Tooth Coronal Index (TCI) method by Jain et al. (2018) and Gotmare et al. (2019) offers cost-effective age estimation via radiovisiography imaging [18,19]. Advances in artificial intelligence (AI), as reported by Zheng et al. (2020), enhance dental age analysis [20], while Pires et al. (2021) highlighted challenges in applying Kvaal’s method across diverse populations, requiring refinement [21]. Considering the various methods of age estimation, one comprehensive classification categorizes these methods based on the developmental stages of age estimation, i.e., prenatal, adolescent, and adult stages. After the completion of permanent dentition (ages 17-21), radiographic methods become more challenging. Hence, this study aims to assess the age by calculating the pulp-to-enamel volume ratio of maxillary and mandibular first and second molars using 3D imaging and the TCI method on panoramic radiographs. The goal is to evaluate the reliability and validity of both 3D imaging and the TCI method for accurate age estimation.

## Materials and methods

This comparative, analytical, prospective study was conducted in the Department of Oral Medicine and Radiology over three years from October 2019 to October 2021. The study focused on methods for age estimation through the analysis of dental anatomical structures using radiographs. Subjects between the ages of 16 and 65 years visiting the hospital were enrolled. The study aimed to compare two age estimation techniques frequently used in forensic investigations based on radiographic evaluation. A total of 640 images were scanned, with 320 images analyzed for each method. The participants were categorized into five groups, each having a range of 10 years, for example, the age range of 15 to 25 years, 26 to 35 years, 36 to 45 years, and so on. Inclusion criteria for the study required participants to have subgingival margin level 3, no dental caries, no pulpal calcifications, and clear panoramic and CBCT imaging without artifacts from adjacent metal restorations. Subjects with carious first and second molars, systemic illnesses, or artifacts due to metal restorations were excluded, as were those younger than 16 years or older than 65 years. During the study, subjects were allowed to withdraw at any time if they chose not to continue participation.

Simple random sampling was used to select patients from the Department of Radiology who met the inclusion criteria. The sample size was calculated using the estimated kappa coefficient, relative error, and the difference between the overall agreement probability and the chance agreement probability while keeping power at 80%, type I error at 5%, and type II error at 20%. Approximately 156 observations were included. To account for potential follow-up loss or non-response, 160 males and 160 females were included. The two methods assessed were the TCI method, which utilizes a 2D technique, and the evaluation of the pulp-to-enamel volume ratio using CBCT scans, enhanced with 3D slicer software.

Tooth Coronal Index method

A total of 320 images (160 males, 160 females) were analyzed using the Kodak 9000 C 3D Unit, operated at 70-80 kVp, 10 mA, with an image acquisition time of 9-10.8 seconds. The cervical line (CL) was drawn from the cementoenamel junction, connecting the mesial and distal dentinoenamel junction (DEJ) points and dividing the tooth into crown and root for measurement.

**Figure 1:**
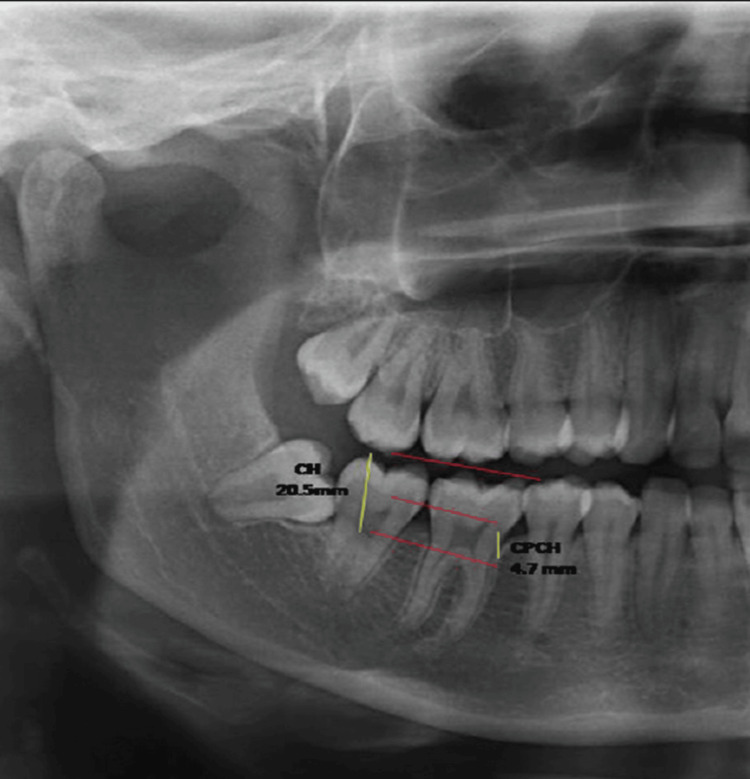
Measurement of the Tooth Coronal Index. The yellow line denotes the height of the crown. The red line denotes the height of the coronal pulp cavity.

The following measurements were obtained from the maxillary first (MX1) and second molars (MX2) and the mandibular first (MND1) and second (MND2) molars: crown height (CH) was the distance from the CL to the highest cusp [22], while coronal pulp cavity height (CPCH) was measured from the CL to the tip of the highest pulp horn [23]. The TCI was calculated as TCI = CPCH × 100/CH (Figure 1). Orthopantomograms, focusing on molars due to anterior teeth blurring, were analyzed to assess TCI, with projection limitations restricting the angle of measurement [24].

Age estimation by evaluating the ratio of pulp-enamel volume on cone-beam computed tomography scans enhanced by 3D Slicer software

**Figure 2:**
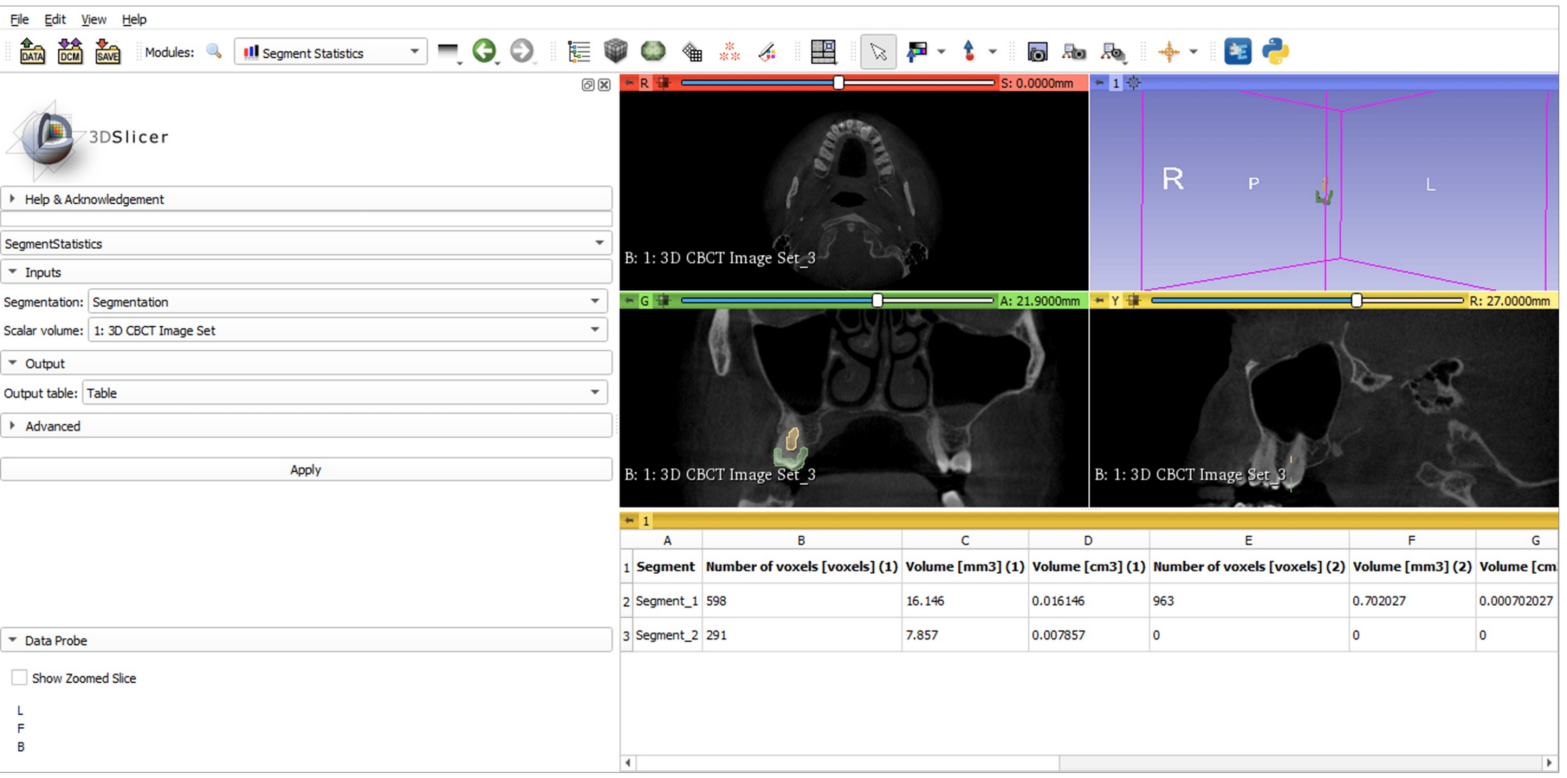
Creating segments of enamel and pulp cavity and construction of a 3D model using the Segment Editor option in 3D Slicer software. Segment 1 (green) denotes the enamel. Segment 2 (yellow) denotes the pulp cavity.

A total of 320 CBCT images between the samples containing maxillary/mandibular first and second molars of the right and left side of 160 males and 160 females referred to the Department of Oral Medicine and Radiology were examined. All images were obtained for diagnosis and treatment, with no unnecessary exposures. CBCT scans were performed using the Carestream Kodak 9000 C 3D Unit, following standard patient positioning and exposure protocols to ensure safety and accuracy as follows: field of view, voxel size: 76.5 × 76.5 × 200 μm, current: 2-15 mA, exposure time: 9-10.8 seconds. Each scan was analyzed in axial, sagittal, and coronal sections for the maxillary and mandibular first and second molars. The largest axial area in the pulp cavity was selected. Images were exported in DICOM format using 3D Slicer software for 3D modeling and volume measurement, with a DICOM database created (Figure 2). The grow cut effect image segmentation algorithm was used to segment the reconstructed 3D models of enamel and pulp cavities. Lines were drawn using the segment editor to identify specific areas of interest in CBCT images, focusing on the layer with the largest axial area in the pulp cavity and enamel. The contrasts highlighted by the lines helped construct 3D models of the pulp cavity and enamel. Any incomplete or excessive areas were repaired layer by layer before calculating pulp volume (PV), enamel volume (EV), and their ratio (PV/EV). Segment statistics in the software were used for these calculations.

Data were entered into a computer using a coding system and checked for entry errors. The data were compiled using MS Office Excel version 2019 (Microsoft Corp., Redmond, WA, USA) and subjected to statistical analysis using SPSS version 26.0 (IBM Corp., Armonk, NY, USA). Descriptive statistics, including frequencies and percentages for categorical data, and means and standard deviations (SDs) for numerical data, were calculated. For comparisons between two groups, the t-test was used, while one-way analysis of variance (ANOVA) was applied for comparisons among more than two groups. Pearson’s correlation coefficient was used to assess bivariate correlations, and regression analysis was conducted to evaluate relationships between numerical variables. For all statistical tests, p-values <0.05 were considered statistically significant, keeping α error at 5% and β error at 20%, leading to a study power of 80%.

## Results

This study evaluated two age estimation methods using 320 teeth (80 from each molar type) from subjects aged 15 to 65 years, categorized into five age groups. The data for maxillary and mandibular first and second molars were statistically analyzed. A statistically non-significant correlation was observed among all variables (p > 0.05), except for MX2, which demonstrated a significant correlation with age. MX2 was found to be a reliable predictor for the relationship between TCI and age (Appendix 1). The standard error of the estimate between actual and predicted age was 14.57, with age considered as the dependent variable and TCI MND2, TCI MX1, TCI MX2, and TCI MND1 as independent variables.

A linear regression model was applied, resulting in the following equation:

$\text{Age=83.244+(-0.088&times;TCIMX1)+(-0.239&times;TCIMX2)+(0.025&times;TCIMND1)+(0.071&times;TCIMND2)}$

Regression analysis revealed an R² value approaching 0, indicating a poor model fit with low predictive reliability for population-wide generalization (Table 1).

**Table 1:** Coefficients and model summary for all four teeth. TCI = Tooth Coronal Index; MX = maxilla; MND = mandible

Model	Unstandardized coefficients		Standardized coefficients	Sig.	R^2^	Adjusted R^2^	Standard error of the estimate
	B	Standard error	Beta				
(Constant)	83.244	31.824	-	-	-	-	-
TCI MX1	-0.088	0.092	-0.107	0.0110	0.00	0.00	14.870
TCI MX2	-0.239	0.108	-0.250	0.0236	0.44	0.44	14.470
TCI MND 1	0.025	0.096	0.030	0.012	-0.013	-0.013	14.890
TCI MND 2	0.071	0.074	0.107	0.073	-0.007	-0.007	14.852

The standard error of estimate between actual and predicted age for each tooth was as follows: 14.870 (MX1), 14.470 (MX2), 14.890 (MND1), and 14.852 (MND2). Internal consistency was evaluated using Cronbach’s alpha, which demonstrated an acceptable to good reliability (α = >0.7 to 0.8), except for CH MX2 (Table 2). A moderate-to-strong agreement was observed (single measures intraclass coefficient = >0.5), with a statistically highly significant p-value (p < 0.01). No statistically significant differences were noted for intergroup comparisons of numerical variables across age groups (p > 0.05).

**Table 2:** Intraclass correlations and Cronbach’s alpha. ** = statistically highly significant difference (p < 0.001). CH = crown height; CPCH = coronal pulp cavity height; MX = maxilla; MND = mandible

Variable	Cronbach’s alpha	Intraclass correlation		Lower bound	Upper bound	Value	P-value
CPCH MX1	0.912	Single measures	0.83	0.75	0.89	11.31	0.000**
		Average measures	0.91	0.86	0.94	11.31	0.000**
CH MX1	0.840	Single measures	0.72	0.60	0.81	6.24	0.000**
		Average measures	0.84	0.75	0.89	6.24	0.000**
CPCH MX2	0.920	Single measures	0.85	0.77	0.90	12.45	0.000**
		Average measures	0.92	0.87	0.94	12.45	0.000**
CH MX2	0.696	Single measures	0.53	0.35	0.67	3.28	0.000**
		Average measures	0.69	0.52	0.80	3.28	0.000**
CPCH MND1	0.899	Single measures	0.81	0.72	0.87	9.88	0.000**
		Average measures	0.89	0.84	0.93	9.88	0.000**
CH MND1	0.868	Single measures	0.76	0.65	0.84	7.55	0.000**
		Average measures	0.86	0.79	0.91	7.55	0.000**
CPCH MND2	0.859	Single measures	0.75	0.64	0.83	7.10	0.000**
		Average measures	0.85	0.78	0.91	7.10	0.000**
CH MND2	0.84	Single measures	0.73	0.61	0.081	6.45	0.000**
		Average measures	0.84	0.75	0.90	6.45	0.00**

The mean PV/EV for different age groups were as follows: for maxillary first molar in the age groups 1, 2, 3, 4, and 5: 192.43 ± 23.52, 191.20 ± 19.93, 183.55 ± 18.61, 180.24 ± 13.15, and 189.48 ± 11.031, respectively. Other tooth values are listed in Appendix 2. Intergroup comparisons between investigators showed no statistically significant differences (p > 0.05) for TCI MX1, TCI MX2, TCI MND1, and TCI MND2 (Table 3). A Pearson’s correlation analysis revealed a statistically highly significant positive correlation (p < 0.01) between age and MX1 PV/EV, MX2 PV/EV, and MND2 PV/EV. A statistically significant positive but moderate correlation (p < 0.01) was observed between age and MND1 PV/EV (Appendix 3).

**Table 3:** Intergroup comparison of values between the investigators. TCI = Tooth Coronal Index; MX = maxilla; MND = mandible

Investigator		Number of samples	Mean	Standard deviation	Standard error of mean	t value	P-value
TCI MX1	1	80	187.49	17.98	2.01	0.17	0.86
	2	80	186.98	19.30	2.15		
TCI MX2	1	80	187.02	15.49	1.73	0.11	0.90
	2	80	186.72	18.32	2.04		
TCI MND 1	1	80	189.14	17.56	1.96	0.65	0.51
	2	80	187.32	17.25	1.92		
TCI MND 2	1	80	191.90	22.384	2.50	1.64	0.10
	2	80	186.36	20.3162	2.27		

A linear regression model was applied using age as the dependent variable and MND2 PV/EV, MND1 PV/EV, MX1 PV/EV, and MX2 PV/EV as predictors. The R² value >0.8 indicated strong external validity. The standard error of estimate between actual and predicted age was 4.34 (Appendix 3).

The regression equation derived was: 

$Age=&minus;5.297+(74.932&times;MX1 PV/EV)+(200.539&times;MX2 PV/EV)&minus;(41.874&times;MND1 PV/EV)+(22.681&times;MND2 PV/EV)$

Using standardized coefficients: 

$Age=0.297&times;MX1 PV/EV+0.620&times;MX2 PV/EV$

Further regression analysis showed R² values nearing 1, indicating a strong predictive model (Table 4). The standard error of estimate between actual and predicted age for all four teeth was 7.19, 4.95, 12.52, and 6.92, respectively. A statistically highly significant difference (p < 0.01) was noted between groups for all variables. The youngest subject in the study was 16 years old, while the oldest was 64 years old. The mean PV/EV for different age groups is provided in Appendix 4.

**Table 4:** Determination coefficients and model summary for all four teeth. MX = maxilla; MND = mandible; PV = pulp volume; EV = enamel volume

	Unstandardized coefficients		Standardized coefficients		95% confidence interval		R^2^	Adjusted R^2^	Standard error of the estimate
	B	Standard error	Beta	Sig.	Lower bound	Upper bound			
(Constant)	-5.29	3.84	-	0.17	-12.95	2.36	-	-	-
MX1 PV/EV	74.93	15.67	0.29	0.00	43.70	106.15	0.76	0.75	7.19
MX2 PV/EV	200.53	29.85	0.62	0.00	141.06	260.00	0.88	0.88	4.95
MND1 PV/EV	-41.87	31.58	-0.05	0.18	-104.80	21.05	0.27	0.27	12.52
MND2 PV/EV	22.68	15.95	0.11	0.15	-9.09	54.46	0.78	0.77	6.92

## Discussion

Unlike bones, teeth remain intact after body decomposition and are less affected by diseases or drug intake, making them ideal for forensic and archaeological investigations [3,25]. Age estimation in dentistry includes methods, such as morphological, histological, and biochemical assessments, which may require tooth extraction [3]. Radiographic techniques, however, allow non-invasive age estimation using morphometric evaluation, making them ideal for living individuals [1,26]. Forensic odontologists initially used Gustafson’s method of macrostructural changes for age estimation [2]. Bodecker later established the correlation between secondary dentin apposition and age, particularly in molars [27]. While 2D radiographs were used to calculate pulp/tooth ratios, they had limitations in measuring 3D structures accurately [11,28]. The advent of CBCT has improved precision in age estimation by overcoming the limitations of 2D imaging [12]. Hence, this study aimed to evaluate age by calculating the ratio of pulp/enamel volume on maxillary/mandibular first and second molars using 3D imaging and using the TCI method on panoramic radiographs and assess the reliability and the validity of both methods in age assessment.

The use of digital panoramic and CBCT radiographs in this study offered advantages such as minimal time, repeatability, reduced radiation exposure, and improved accuracy [13]. Radiographs were obtained from patients in an institutional setting, likely over-representing lower socioeconomic groups. The small sample size and heterogeneity posed challenges, reducing the accuracy for age and sexual dimorphism. To mitigate these effects, a total of 320 teeth were examined by two investigators to ensure thoroughness and reliability in the study. Maxillary and mandibular first and second molars were examined using panoramic radiographs to avoid intraoral films affected by pathologies or restorative materials. Panoramic radiographs helped select the teeth with clear pulp chambers, while CBCT enabled volumetric analysis of enamel and pulp. A comparison was made to determine the best method for forensic age assessment. The TCI, a morphometric assessment using the radiographs of human teeth, was originally developed by Ikeda et al. in 1985, where the length of the coronal pulp and crown were measured and TCI was assessed using the prints of radiographs of extracted human teeth. The correlation coefficients ranged from -0.73 (female molars) to -0.89 (female premolars), and no significant differences were found in age estimation using gender-specific formula [23]. Drushni applied the TCI after Ikeda et al. in a sample of 846 intact teeth of known age and gender using panoramic radiographs. The coefficient ranged from -0.92 to -0.87, with the range from 5.88 to 6.66 years as the standard error [24]. In this study, maxillary and mandibular first and second molars of the right and left side were used to estimate the TCI as the extent of the pulpal chamber is visible in these teeth, which was in accordance with Drusini [24], and Harris and Nortjé [29]. In our study, the correlation was highly negative for all four teeth: MX1 (r = 0.110), MX2 (r = 0.236), MND1 (r = 0.012), and MND2 (r = 0.073).

As the standard error of estimate between actual age and predicted age was very high, the results of this study indicated that the TCI can be used for evaluating dental age in our population; however, it was not completely reliable and cannot be generalized. Intergroup comparison of mean TCI of the total sample was performed using the one-way ANOVA test, which showed that there was a statistically non-significant difference in all four teeth, the intergroup comparison of mean TCI (p = 0.073) with higher values for the age group of the mandibular first molar. In the intergroup comparison, no significant difference was found between males and females for CH and CPCH values, except for CPCH MND2 for investigator 2, where females had higher values. For right versus left side comparisons, significant differences were noted for CH MX2 and CH MX1, with both higher on the left side. The results of this study were in accordance with the results of the previous studies by Badar et al. in a Pakistani sample. They found that the mean correlation coefficient (r) between the chronological age and TCI was -0.27, and a very weak correlation was found between tooth and TCI [30].

The results in this study were also in agreement with a previous study that used multiple regression analysis in a Western Australian population and found that the correlation coefficient was highest for the mandibular right first premolar consistently for the combined sample (r = -0.262). Similar studies done on the Indian population to test the validity of different age estimation formulae have shown results contrary to the current study. A study by Jain et al. in 2017 showed a statistically significant negative correlation between the chronological age and TCI of mandibular first molar (r = -0.178) and second premolar (r = -0.187) [31]. Our results were contrary to the results obtained by Zhang et al. (r = -0.850) [32], and Havale et al. (r = -0.59) [33]. In the epoch of booming technologies, the application of 3D radiology has gained attention. 2D images do not provide entire morphological changes in the teeth and provide a linear measurement which fails to provide any volumetric information regarding the teeth and related structures, whereas, on the other hand, dental CBCT has a larger scanning space, low radioactivity, and no requirement for extraction of teeth. Hence, in this study, a volumetric analysis of teeth was performed using CBCT [34].

After the eruption of a tooth, the formation of secondary dentine leads to a decrease in pulp volume. This change can be considered a valuable marker in the estimation of age for adults and measured from dental radiographs [1,35]. Cameriere et al. evaluated the pulp/tooth area ratio which can be considered as an indicator of age [36]. In our study, maxillary/mandibular molars were examined using computer-aided drafting software. The pulp-tooth volume ratio (PV/TV) was calculated to minimize bias from individual variation. Segmentation was done using a growth-cut effect in 3D Slicer software, constructing an enamel-pulp model for accurate age estimation. 3D Slicer version (4.11.2021), a free software, illustrates an innovative application of CBCT image data, which calculates volume and can be helpful in the determination of chronological age. In our study, we tried to verify the usefulness and validity of this software development [20].

The correlation coefficients were MX1 (r = 0.873), MX2 (r = 0.942), MND (r = 0.528), and MND2 (r = 0.883). Using a linear regression model, the equations were derived where age was kept as the dependent variable and PV/EV as the independent variable, the same as PV/EV. The determination coefficients were comparatively high for all four teeth, with MX1 (r^2^ = 0.75), MX2 (r^2^ = 0.88), MND1 (r^2^ = 0.27), and MND2 (r^2^ = 0.78), with the highest for MND1. The results were in agreement with Zheng et al. [20], who used multiple regression analysis in a Chinese population and found a determination coefficient for first molars (r^2^ = 0.74). Similar to our study, Sue et al. in 2017 showed a significant correlation between pulp volume in the Chinese population and age, with an r^2^ of 0.586 for maxillary molars, and an r^2^ of 0.609 for mandibular molars [17]. Most studies conducted in the Indian population are based on the correlation of anterior teeth with age. Our study showed a significant correlation of multirooted teeth in the Indian population. In our study, the standard error of estimate between real age and predicted age for all the teeth was more than five years, except for MX2 (4.952). Hence, MX2 can be reliable in evaluating age. Intergroup comparison of the mean PV/EV of the total sample was done using the one-way ANOVA test, which showed that there was a statistically highly significant difference in the intergroup comparison of the mean (p < 0.001).

Our results were similar to the study findings of Star et al. [12], Jagannathan et al. [28], and Sakuma et al. [14], but differ from those of Someda et al. [37], Porto et al. [38], and Biuki et al. [39]. Factors such as sample size, sample population, and tooth type all contribute to the results. In the intergroup comparison for the side, there was a statistically non-significant difference in PV/EV, except for MND1, where there was a statistically significant difference (p = 0.03), with higher values for the left. A study by Jagannathan et al. [28] did not find any difference on either side in which the PV/TV of canine teeth was compared. Thus, these results indicated that the age-related changes on both sides can be considered the same. Inter-investigator comparison of EV for all four teeth showed a statistically non-significant difference in the values between the observers for all variables, except for mean of observer 1 MX2 (p = 0.02), where a statistically significant difference was seen. Inter-investigator comparison of PV for all four teeth showed a non-significant difference.

The limited sample size, drawn from a specific geographic area, may have contributed to the underestimation of age in some subjects and introduced bias, potentially reducing accuracy in age determination. A larger, more heterogeneous sample would allow for better refinement and optimization of the method. Some other limitations of the study include high standard error, suggestive of poor predictive ability and lack of assessment of intra-observer and inter-observer variability, which could affect the reliability and reproducibility of the method. Additionally, newer CBCT systems, with improved contrast resolution (12 bit, 4,096 Gray levels), could enhance tooth segmentation and improve the precision of age estimation. Further advancements in third-party software for volumetric analysis could also reduce processing time while improving the accuracy and precision of the technique. Future studies can also employ advanced radiographic techniques, such as the fuzzy neural network with teaching learning-based optimization and 4D imaging, which could provide more precise calculations of tooth characteristics such as density, size, and geometric features. Future research should investigate their application in forensic dentistry to enhance accuracy in age estimation.

## Conclusions

The findings of the present study highlight the superiority of 3D imaging over 2D techniques for forensic age estimation, particularly in adult populations. The analysis of maxillary and mandibular molars using volumetric assessment proved more reliable than conventional radiographic methods. While the TCI method offered some correlation with age, it lacked the predictive accuracy necessary for forensic applications. In contrast, the pulp-to-enamel volume ratio derived from CBCT demonstrated a strong and consistent relationship with age, making it a more effective tool for forensic and medicolegal investigations. Despite its advantages, 3D imaging is not without limitations, including accessibility, cost, and the need for advanced computational processing. However, its ability to provide precise volumetric measurements makes it a promising avenue for future research and forensic practice. Refinements in imaging technology and automated analysis techniques could further enhance its applicability, improving the accuracy and efficiency of age estimation. Future studies should explore larger and more diverse populations to validate and optimize these methodologies for widespread forensic use.

## Appendices

 Appendix 1

**Table 5:** Bivariate correlations between actual age and all teeth and model summary and parameter estimates of age (the independent variable is TCI, and the dependable variable is age). * = statistically significant (p < 0.05). TCI = Tooth Coronal Index; MX = maxilla; MND = mandible

		TCI MX1	TCI MX2	TCI MND1	TCI MND2	R^2^	Adjusted R^2^	Standard error of the estimate
Actual age	r	0.034	-	-	-	0.079	0.030	14.574
	p	0.76	-	-	-			
	n	80	-	-	-			
	r	-0.09	0.160	-	-			
	p	0.40	0.15	-	-			
	n	80	80	-	-			
	r	0.079	0.11	0.11	-			
	p	0.48	0.29	0.31	-			
	n	80	80	80	-			
	r	-0.110	-0.236*	0.012	0.073			
	p	0.331	0.035*	0.913	0.522			
	n	80	80	80	80			

 Appendix 2

**Table 6:** Intergroup comparison of numerical variables with age groups. TCI = Tooth Coronal Index; MX = maxilla; MND = mandible

					95% confidence interval for mean		Minimum	Maximum	F value	P-value
	Age group	Mean	SD	SE	Lower bound	Upper bound				
TCI MX1	1	192.43	23.52	5.88	179.89	204.96	159.09	231.42	1.35	0.260
	2	191.20	19.93	4.98	180.58	201.82	152.17	225		
	3	183.55	18.61	4.65	173.63	193.47	160.87	242.85		
	4	180.24	13.15	3.39	172.95	187.52	157.89	203.22		
	5	189.48	11.03	2.67	183.81	195.16	172.97	211.42		
	Total	187.49	17.98	2.01	183.49	191.50	152.17	242.85		
TCI MX2	1	189.74	17.90	4.47	180.20	199.28	166.66	231.42	1.61	0.179
	2	190.86	16.35	4.08	182.14	199.58	163.63	221.87		
	3	189.84	18.22	4.55	180.13	199.55	160.00	230.00		
	4	185.70	19.88	2.55	180.23	191.18	170.27	203.33		
	5	179.36	11.99	2.90	173.20	185.53	157.89	200.00		
	Total	187.02	15.49	1.73	183.57	190.47	157.89	231.42		
TCI MND1	1	18.02 5	4.50	175.15	194.36	162.22	222.85	184.76	2.238	0.073
	2	23.31	5.82	177.53	202.38	157.89	238.23	189.96		
	3	16.60	4.15	190.03	207.73	171.05	229.41	198.88		
	4	12.86	3.32	174.90	189.15	164.10	200.00	182.02		
	5	11.98	2.90	183.44	195.76	174.28	210.00	189.60		
	Total	17.56	1.96	185.23	193.0	157.89	238.23	189.14		
TCI MND2	1	189.81	24.71	6.178	176.64	202.98	155.556	225.000	1.12	0.349
	2	184.50	17.24	4.310	175.31	193.69	155.55	215.62		
	3	196.05	20.46	5.11	185.15	206.96	162.79	225.00		
	4	199.89	32.34	8.35	181.97	217.80	172.22	310.00		
	5	189.87	13.18	3.197	183.09	196.65	170.27	212.93		
	Total	191.90	22.38	2.50	186.92	196.88	155.55	310.00		

Appendix 3

**Table 7:** Bivariate correlations of PV/EV ratio with age for all four teeth along with model summary and parameter estimates. MX = maxilla; MND = mandible; PV = pulp volume; EV = enamel volume

	Actual age		R^2^	Adjusted R^2^	Standard error of the estimate
MX1 PV/EV	r	-	0.91	0.91	4.34
	p	-			
	n	80			
MX2 PV/EV	r	0.94			
	p	0.00			
	n	80			
MND1 PV/EV	r	0.52			
	p	0.00			
	n	80			
MND2 PV/EV	r	0.88			
	p	0.00			
	n	80			

 Appendix 4

**Table 8:** Intergroup comparison of all four teeth with age groups for total sample. ** = statistically significant (p < 0.001). MX = maxilla; MND = mandible; PV = pulp volume; EV = enamel volume; ANOVA = analysis of variance

Age group						95% confidence interval for mean				F value	P-value of one-way ANOVA
		N	Mean	SD	SE	Lower bound	Upper bound	Minimum	Maximum		
MX1 PV/EV	1	16	0.100	0.007	0.001	0.09	0.10	0.08	0.10	123.39	0.00**
	2	16	0.12	0.018	0.004	0.11	0.13	0.08	0.14		
	3	16	0.13	0.010	0.002	0.13	0.14	0.12	0.15		
	4	16	0.17	0.020	0.005	0.16	0.18	0.12	0.20		
	5	16	0.25	0.037	0.009	0.23	0.27	0.18	0.31		
MX2 PV/EV	1	16	0.11	0.011	0.002	0.10	0.11	0.09	0.13	234.37	0.00**
	2	16	0.13	0.015	0.003	0.13	0.14	0.11	0.16		
	3	16	0.18	0.008	0.002	0.18	0.19	0.16	0.19		
	4	16	0.20	0.017	0.004	0.19	0.21	0.17	0.23		
	5	16	0.22	0.008	0.002	0.22	0.23	0.20	0.24		
MND1 PV/EV	1	16	0.12	0.007	0.001	0.11	0.12	0.10	0.130	13.51	0.00**
	2	16	0.13	0.016	0.004	0.12	0.14	0.11	0.17		
	3	16	0.14	0.017	0.004	0.140	0.15	0.13	0.18		
	4	16	0.013	0.013	0.003	0.13	0.14	0.12	0.18		
	5	16	0.15	0.018	0.004	0.14	0.16	0.13	0.21		
MND2 PV/EV	1	16	0.12	0.012	0.003	0.12	00.13	0.10	0.14	130.10	0.00**
	2	16	0.15	0.00	0.001	0.14	0.15	0.14	0.16		
	3	16	0.24	0.053	0.013	0.21	0.27	0.13	0.29		
